# Systemic sclerosis and anorectal dysfunction: The Leeds experience

**DOI:** 10.1177/23971983241241203

**Published:** 2024-04-08

**Authors:** Nikhil Suresh, Ranjitha Karanth, Ramsah Cheah, John Casey, David G Jayne, Francesco Del Galdo

**Affiliations:** 1 Leeds Institute of Rheumatic and Musculoskeletal Medicine, University of Leeds, Leeds, UK; 2 NIHR Biomedical Research Centre, Leeds Teaching Hospitals NHS Trust, Chapel Allerton Hospital, Leeds, UK

**Keywords:** Systemic sclerosis, anorectum, internal anal sphincter, faecal incontinence

## Abstract

Systemic sclerosis is an autoimmune disorder which frequently affects the gastrointestinal tract. Anorectal dysfunction is common in systemic sclerosis and is manifested mainly by atrophy of internal anal sphincter. Faecal incontinence is the result of internal anal sphincter atrophy secondary to systemic sclerosis. In this study, we aimed to assess the internal anal sphincter in 17 patients with faecal incontinence and systemic sclerosis using anorectal manometry and endoanal ultrasound and compare them with an age and gender-matched control group without systemic sclerosis. Most patients have limited cutaneous systemic sclerosis. Majority of the patients with systemic sclerosis and faecal incontinence presented with symptoms of faecal leakage and urgency. Systemic sclerosis patients had low basal sphincter pressures. The mean thickness of internal anal sphincter in systemic sclerosis group was significantly lower than the control group (p < 0.001). Rectal sensation is preserved in systemic sclerosis. There was no difference in the mean thickness of the external anal sphincter between the two groups. To conclude internal anal sphincter is atrophic in systemic sclerosis resulting in decreased resting sphincter pressures and passive faecal leakage. Further investigations and studies are needed to determine the natural course of faecal incontinence in systemic sclerosis, associated risk factors and efficacy of therapeutic interventions.

## Key message

**What is already known on this topic:** Anorectal dysfunction is a common gastrointestinal manifestation in systemic sclerosis characterised by selective atrophy of internal anal sphincter resulting in faecal incontinence.

**What this study adds:** Faecal incontinence is predominantly seen in patients with limited cutaneous systemic sclerosis and characterised by low-resting pressures in the anorectal sphincter complex.

**How this study might affect research and practice:** A large collaborative multicentre cohort study is needed to explore these findings and treatment options. Our study highlights the needs for early recognition of anorectal symptoms in patients with limited scleroderma. This might enable us to decrease the morbidity associated and improve associated quality of life in patients with scleroderma associated faecal incontinence.

## Introduction

Systemic sclerosis (SSc) is an autoimmune rheumatological disease characterised by a chronic autoimmune inflammatory process leading to an increased deposition of collagen and extra cellular matrix in the skin, internal organs, and blood vessels.^1–3^ Gastrointestinal involvement in scleroderma is manifested by smooth muscle atrophy, while the aetiology for which is only partially understood.^2–5^ The British Society of Rheumatology (BSR) has stressed the importance of early recognition and diagnosis of gastrointestinal involvement and appropriate referral to a specialist systemic sclerosis centre. Gastrointestinal involvement is estimated to result in hospitalisation in approximately 15% of cases.^6^ Oesophageal involvement is common, causing dysmotility and gastro-oesophageal reflux disease (GERD) in up to 90% of the patients, but any other part of the gastrointestinal tract can be affected.^7–10^

The Association of Coloproctology of Great Britain and Ireland (ACPGBI) defines faecal incontinence (FI) as the uncontrollable loss of solid or liquid stool or the loos of wind (flatus) with leakage. It is reported in up to 0.9% of adults between 40 and 65 years and increases to 2.4% above 65 years. It has a significant impact on quality of life due to its physical, social, and psychological impact.^4^ Obstetric injury is the most common cause of FI in western populations. Compared to the general population, the incidence of FI in SSc is much higher, with rates estimated to be between 20% and 40%, but it is probably under reported due to the reluctance of patients to discuss embarrassing topics.^6,8,11–14^ In the anorectum, the internal anal sphincter (IAS) is the structure mainly affected by SSc, and it is thought to be the main contributor to symptoms of FI.^2–4,8,11,13,15–22^

Clinical investigations for FI include anorectal manometry and endoanal ultrasound (EAUS) to assess sphincter function and structure respectively. There is little data available on the manometric and ultrasound findings that underlie SSc involvement and to guide therapy.

To address this knowledge gap, we aimed to compare anorectal manometry and EAUS findings in a cohort of SSc patients suffering from FI with an age and gender-matched cohort of patients with FI without SSc.

## Material and methods

### Patients

Seventeen consecutive patients with symptoms of FI were identified within the observational cohort STRIKE (Stratification for Risk of Progression in Scleroderma, Ethical approval 2014-2027, IRAS 178638) database of patients being treated at Leeds Teaching Hospitals NHS Trust between December 2014 and October 2021. These patients were assessed for symptom using the gastrointestinal visual analogue score (GI VAS). Patients included in this study attended the Gastrointestinal Physiology Unit at Leeds Teaching Hospitals NHS Trust for anorectal manometry and EAUS, according to standard of care diagnostic workup of FI. A retrospective gender and age-matched cohort of patients without SSc undergoing investigations for FI was anonymously selected from the gastrointestinal physiology database. Patients with significant pelvic floor dysfunction secondary to vaginal births or other causes like history of perineal injury, perineal surgery and previous rectal surgery were excluded from the SSc cohort.

### Anorectal manometry

Conventional anorectal manometry (2013–2014) or high-resolution anorectal manometry (2015 onwards) was performed using a 10-channel single-use water-perfused catheter with channels spaced at 8 mm intervals from the tip (Mui, Canada), according to the Leeds Teaching Hospital Trust standard of care procedures. The recordings were analysed using Solar GI Manometry system (Laborie Medical Technologies, Portsmouth, NH, USA). Normal values were determined using the international anorectal physiology working group (IAPWG) recommendations.^23^ Basal sphincter pressure (normal = 34–101 mm Hg) and incremental squeeze pressure (normal > 27 mm Hg) were measured. The difference between these two variables was calculated as the incremental maximum squeeze pressure (normal > 27 mm Hg). Rectal sensory testing to distension was performed using a rectal balloon placed 3–5 cm above the anorectal junction, and the first constant sensation volume (FCSV), desire to defecate volume (DDV), and maximum tolerated volume (MTV) recorded.

### EAUS

Representative two-dimensional cross-sectional axial images of the anal canal from the level of puborectalis through to the anal verge were captured using a 10-MHz transducer (Hitachi EUP-R54AW, Hitachi Medical Systems, Twinsburg, OH, USA). The thicknesses of the internal and external anal sphincter (IAS and EAS) were measured at the 3 and 9 o’clock positions.

#### Statistics

The two groups were analysed for categorical data using χ^2^ or Fisher exact tests. Continuous data were presented either by means for parametric data set or medians for non-parametric data set. The continuous data were analysed using the Mann–Whitney *U*-test for non-parametric data. Differences between the two groups were deemed to be significant if the p-value was <0.05.

## Results

### Patient demographics

The demographics and clinical characteristics of the scleroderma and control cohorts are shown in Table 1. All patients were female. There was no difference in the mean age of the two cohorts (SSc 61.2 years vs control 61.6 years; p = 0.901). A total of 17 patients with SSc were included in the study. Fourteen patients in the SSc group had limited cutaneous systemic sclerosis (14/17; 82.4%), with two having diffuse cutaneous systemic sclerosis (11.8%) and 1 patient with SSc overlap (0.058%). Most of the patients in our SSc cohort were positive for anti-centromere antibody (ACA; 75%) and antinuclear antibody (ANA; 82.4%). The median duration of SSc in the study group was 14.3 years. In comparison to the control group, patients with SSc were more likely to present with symptoms of faecal leakage (23.5% vs 9.1%; p = 0.163) and increased bowel frequency (17.6% v 3.0%; p = 0.071) and less likely to suffer from obstructed defaecation symptoms (11.8% vs 30.3%; 0.146). 12% of patients presented with mixed symptoms like passive leakage and urgency.

**Table 1.** Demographics and clinical characteristics of the scleroderma cohort.

	FI (Control) n = 33	Scleroderma n = 17	p-value
Basic demographics			
Age (when physiological tests were done), mean (95% CI)	61.6 (57.9–65.4)	61.2 (54.2–68.2)	0.901
Gender (female), n (%)	33/33 (100)	17/17 (100)	–
Symptom presentations			
Faecal incontinence, n (%)	33/33 (100)	17/17 (100)	–
Faecal urgency, n (%)	15/33 (45.5)	3/17 (17.6)	0.052
Faecal leakage, n (%)	3/33 (9.1)	4/17 (23.5)	0.163
Increased bowel frequency, n (%)	1/33 (3.0)	3/17 (17.6)	0.071
Obstructive defaecation, n (%)	10/33 (30.3)	2/17 (11.8)	0.146
Constipation, n (%)	10/33 (30.3)	1/17 (5.9)*	0.048
Diarrhoea/loose stool, n (%)	8/33 (24.2)	3/17 (17.6)	0.594
Scleroderma diagnosis			
Limited cutaneous systemic sclerosis, n (%)		14/17 (82.4)	
Diffuse cutaneous systemic sclerosis, n (%)		2/17 (11.8)	
SSc overlap		1/17 (5.9)	
Antibody profile			
Anti-centromere antibodies (ACA), n (%)		12/16 (75.0)	
Antinuclear antibodies (ANA), n (%)		14/17 (82.4)	
Serum anti-topoisomerase (SCL-70), n (%)		2/13 (15.4)	

FI: faecal incontinence; CI: confidence interval. *p < 0. 05

### Anorectal manometry

**Figure 1.**
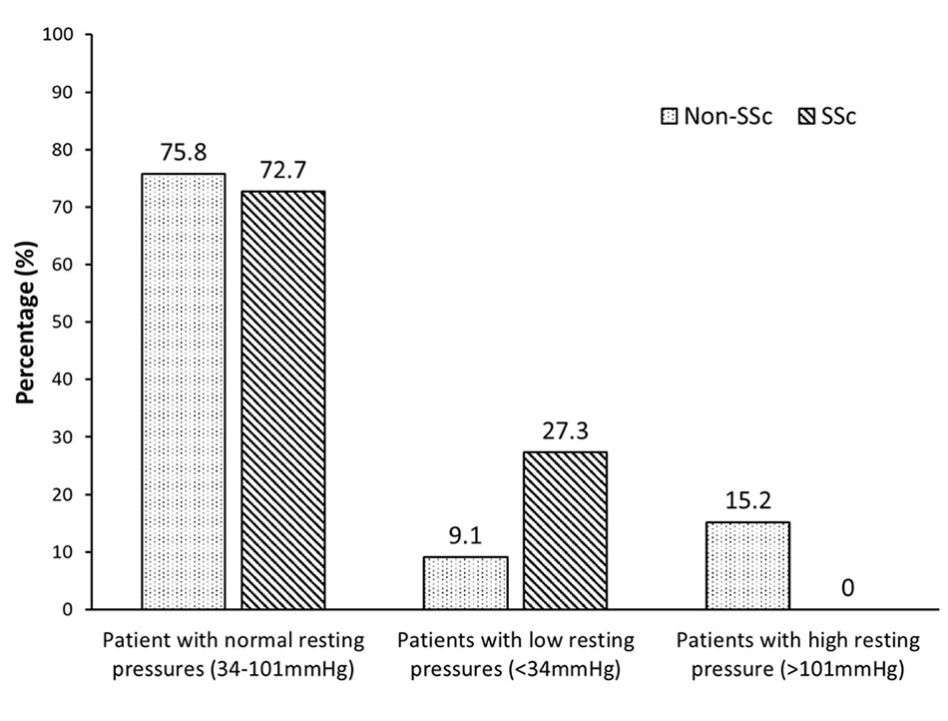
Percentage of patients with normal (34–101 mm Hg), low (<34 mm Hg) and high (>101 mm Hg) resting pressures between in patients with scleroderma (SSc) and FI and non-scleroderma (non-SSc) patients with FI. FI: faecal incontinence.

**Figure 2.**
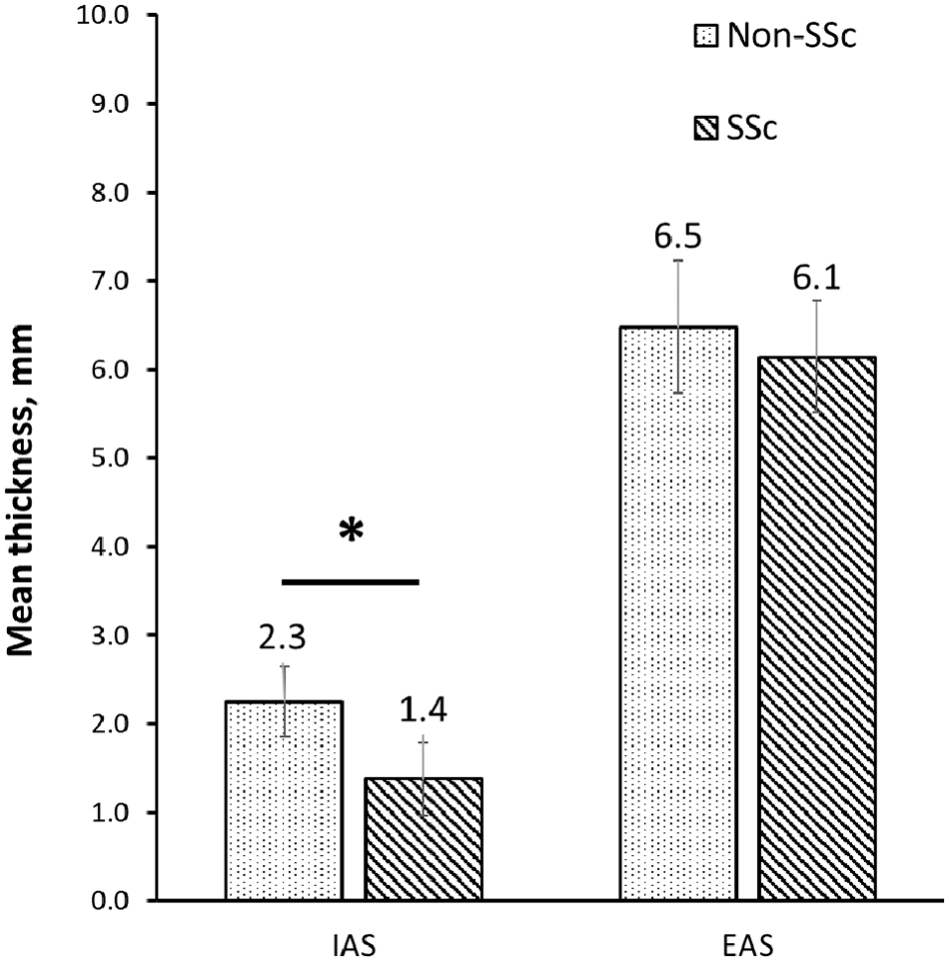
Mean thickness of IAS and EAS between patients with scleroderma (SSc) and FI and non-scleroderma (non-SSc) patients with FI. Error bars indicate the standard deviation of the data. IAS: internal anal sphincter; EAS: external anal sphincter. *p < 0.05.

The SSc cohort had significantly lower basal sphincter pressures than patients with FI and no SSc (control) (SSc median (interquartile range (IQR)) = 39.7 mm Hg (33–56) vs control 70.0 mm Hg (49–93) p = 0.0045) (Table 2). Although the proportions of patients with normal anal resting pressures, as assessed with the London Classification, were similar between groups (75.8% vs 72.7%), 27.3% of SSc patients had lower-resting pressures (resting pressures of <34 mm Hg) as compared with only 9.1% in the control group (Figure 1). There was 15.2% of the control patients with high anal resting pressure (resting pressures of >101 mm Hg), and none for the SSc group (Table 2 and Figure 2). There were no statistically significant differences in maximum squeeze pressures (SSc median (IQR) = 82 mm Hg (72–107) vs control 108 mm Hg (95–164) p = 0.110) and maximum incremental squeeze pressures (SSc median (IQR) = 44 mm Hg (41–61) vs control 35 mm Hg (26–124) p = 0.735) between the two groups. There was no significant difference in the sensory values between SSc patients and control patients: FCSV (SSc median (IQR) = 33.5 mL (28–48) vs control 32 mL (26–64) p = 0.897), DDV (SSc 79 mL (58–82) vs control 70 mL (42–108) p = 0.656), and MTV (SSc 114 mL (103–162) vs control 121 mL (67–195) p = 0.734).

**Table 2.** Anorectal manometry and EAUS findings in patients with scleroderma (SSc) and FI and non-scleroderma (non-SSc) patients with FI.

	FI (control) n = 33	Scleroderma n = 17	
Anorectal manometry			
Anal sphincter pressure			
Resting anal sphincter pressure, mm Hg, median (IQR)	70.0 (49.0–93.0)	39.7 (32.9–55.7)*	**0.005**
Patients with normal anal resting tone, n (%)	25/33 (75.8)	8/11 (72.7)	
Patients with very low anal resting pressure (<34 mm Hg), n (%)	3/33 (9.1)	3/11 (27.3)	
Patients with high anal resting pressure (>101 mm Hg), n (%)	5/33 (15.2)	0/11 (0)	0.159
Maximum squeeze pressure, mm Hg, median (IQR)	108.0 (95.0–164.0)	82.0 (72.0–107.1)	0.110
Maximum incremental squeeze pressure, mm Hg, median (IQR)	35.0 (26.0–124. 0)	44.0 (41.0–61.0)	0.735
Rectal sensation			
FCSV, mL, median (IQR)	32.0 (26.0–64.0)	33.5 (28.0–48.0)	0.897
DDV, mL, median (IQR)	70.0 (42.0–108.0)	79.0 (58.0–82.0)	0.656
MTV, mL, median (IQR)	121.0 (67.0–195.0)	114.0 (103.0–162.0)	0.734
Endoanal ultrasound			
Mean IAS thickness, mm, mean (95% CI)	2.3 (2.1–2.4)	1.4 (1.1–1.7)*	0.000
Mean EAS thickness, mm, mean (95% CI)	6.5 (6.2–6.8)	6.2 (5.8–6.7)	0.326
Patients with IAS defects (i.e. divided and disruptions), n (%)	6/33 (18.2)	0/9 (0)	0.167
Patients with EAS defects (i.e. disruptions), n (%)	3/33 (9.1)	2/9 (22.2)	0.281

FI: faecal incontinence; IQR: interquartile range; FCSV: first constant sensation volume; DDV: desire to defecate volume; MTV: maximum tolerated volume; IAS: internal anal sphincter; CI: confidence interval; EAS: external anal sphincter.

* p < 0. 05. The significance of bold value p < 0. 05.

### EAUS

The mean thickness of the IAS in the SSc group was significantly lower than controls (SSc 1.4 mm (1.1–1.7) vs controls 2.3 mm (2.1–2.4) p < 0.001) (Table 2). There was no difference in the mean thickness of the external anal sphincter between the two groups (SSc 6.2 mm (5.8–6.7) vs control 6.5 mm (6.2–6.8) p = 0.326). The proportions of patients identified with IAS defects, including divided and disruptions of the muscle layer, and with EAS disruptions were not significantly different between the two study groups, as shown in Table 2.

## Discussion

Anorectal involvement is reported in up to 70% of patients with SSc,^2,3,5,13,24^ although FI or soiling is frequently underreported due to the embarrassing nature of the condition. FI is estimated to affect 20%–70% of patients with SSc.^3,8–11,17,25^ Previously studies have investigated anorectal symptoms in small cohort of patients and found that the IAS is usually affected resulting in lower resting pressures, some of the findings like rectal sensation, compliance, and antibody profile in SSc are variably reported which could be explained due to the small sample sizes of the existing studies.^2,3,11–13,15,16,19,20,26^ The multifactorial pathophysiology underlying FI in SSc has previously been reviewed and recommendations made for earlier screening for FI in patients reporting gastrointestinal symptoms.^27^ Most patients with SSc and FI have normal resting pressures, although approximately 23% have poor resting pressures due to atrophy of the IAS.^27^ In the non-SSc gro-up, the main cause for FI was external anal sphincter injury during childbirth. Classification of SSc patients into normal and low resting pressures groups might help in stratifying therapy.

In this study, we have evaluated the presenting symptoms, anorectal motility, myogenic response, and sensation in 17 patients with FI and SSc. We have also comprehensively assessed the structure of the anal sphincter complex. We found that in our cohort, FI predominantly occurred in females with limited SSc. Majority of SSc patients with FI had normal resting tone, but in a proportion of patients, this is significantly reduced with preserved rectal sensation. Although the maximal squeeze pressures in SSc group were lower, there was no statistical difference noted probably due to the small sample size.

The discrepancy between the SSc and control groups in resting pressure was explained by a selective atrophy and fibrosis of the IAS with a largely unaffected external anal sphincter resulting in lower-resting pressures. The majority of SSc patients have a normal resting pressure; however, a subset of patients has a low resting anal pressure. The IAS is a circular rectal smooth muscle layer that functions to control passive continence and prevent FI at rest.^28^ The IAS is responsible for up to 80% of resting anal sphincter pressure and basal tone.^2,3,8,13^ Atrophy of the IAS in patients with SSc causes reduced resting pressures leading to passive FI and faecal leakage. Faecal leakage was seldom reported in the control group. EAS defects during childbirth were similar between the groups and there was no difference in rectal sensation between groups. Anorectal dyssynergia is a difficult condition to diagnose and no one test has been shown to be reliable. More usually, anorectal dyssynergia is diagnosed with a combination of dynamic proctography and anorectal manometry with or without a balloon expulsion test. All these tests are laboratory based and performed under non-physiological conditions. They are heavily influenced by patient compliance and their interpretation is subjective. We feel that trying to deduce anorectal dyssynergia based solely on the anorectal manometry data is unlikely to be informative and would probably be misleading.

The limitations of our study are first it is single centre and being retrospective in nature; it is prone to type 1 errors by not detecting a change when one exists. In addition, the anorectal clinical investigations were limited to patients with reported FI, and therefore, we cannot assess the prevalence of IAS changes in patients who did not report this symptom.

While most symptomatic patients in our cohort exhibited limited SSc and preserved rectal sensation, it would be premature to assert that this represents a universal pattern in the progression of the disease across subgroups. A comprehensive multicentre collaborative study could provide insights and address some of these uncertainties.

To summarise, we have shown that the IAS is atrophic in patients with systemic sclerosis resulting in decrease in resting basal sphincter pressure. The external anal sphincter is largely unaffected by the disease. Passive faecal leakage and urgency were the most common presenting symptoms. The increased prevalence and the peculiar findings of FI in SSc support the notion of FI being an organ manifestation of SSc. Further investigations are warranted to determine the natural history, risk factors and eventual optimal window of opportunity for intervention of this very impactful GI manifestation of disease.
